# Antimicrobial and antioxidant properties of the crude peptide extracts of *Galatea paradoxa* and *Patella rustica*

**DOI:** 10.1186/s40064-015-1266-2

**Published:** 2015-09-17

**Authors:** Lawrence Sheringham Borquaye, Godfred Darko, Edward Ocansey, Emmanuel Ankomah

**Affiliations:** Department of Chemistry, Kwame Nkrumah University of Science and Technology, Kumasi, Ghana

**Keywords:** Bacteria, Fungi, Bioactive compounds, Marine molluscs, Freshwater molluscs

## Abstract

This study evaluated the antimicrobial and antioxidant activities of crude peptide extracted from *Galatea paradoxa* (*G. paradoxa*) and *Patella rustica* (*P. rustica*). The extracts were tested against eight strains of bacteria (*Escherichia coli, Staphylococcus aureus, Bacillus subtilis, Salmonella typhi, Enterococcus feacalis, Klebseilla pneumoniae, Streptococcus pneumoniae, Pseudomonas aeruginosa*) and one strain of fungi (*Candida albicans*) using agar well diffusion and broth dilution assays. The extracts from *G. paradoxa* demonstrated a high degree of activity against the bacteria strains but were inactive towards the fungus. *P. rustica*, however, showed a markedly higher antifungal activity but little antibacterial effect. The minimum inhibitory concentrations (MIC) of the extracts determined by the broth tube dilution assay were 17 mg/mL of *G. paradoxa* against the entire spectrum of microorganisms tested except for *C. albicans* which was 20 mg/mL. The MIC of the extracts of *P. rustica* was 13 mg/mL against all the strains of microorganisms tested except for *E. feacalis* (17 mg/mL), *K. pneumoniae* (17 mg/mL) and *C. albicans* (13 mg/mL). Antioxidant activity using the 2,2-diphenyl-1-picrylhydrazyl (DPPH) assay showed scavenging ability on the DPPH radical was 56.77 % at 0.39 mg/mL for *G. paradoxa* and 79.77 % at 0.39 mg/mL for *P. rustica*. The study indicates that the crude peptide extracts from the two molluscs have promising antimicrobial and antioxidant activities that can be harnessed as leads for potential bioactive compounds.

## Background

In recent years, there have been a rise in infectious disease cases all over the world. The emergence and/or reemergence of some of these infectious diseases such as the deadly Ebola viral disease (EVD) have had crippling economic and social impacts in countries affected. This, coupled to the fact that drug resistant pathogens are evolving at a much faster rate than new drugs are being discovered, has heightened the need to search for new classes of antimicrobial agents. The indiscriminate use of drugs to manage infectious diseases have not helped the situation. Many scientists and research programs are therefore prospecting for new antimicrobial agents from plants and animal sources.

Marine invertebrates have proven to be rich sources of bioactive compounds with activities ranging from antimicrobial to antitumor (Martins et al. 2014; Leal et al. 2012; Thakur et al. 2005). Due to the fact that they exhibit broad spectrum antimicrobial activity, possess selective toxicities and are less prone to microbial resistance, antimicrobial peptides represent an exciting class of bioactive compounds that could potentially provide major reprieve for mankind in the efforts to curb/control infections. Because marine invertebrates rely solely on innate immune mechanisms for defense, they represent a potentially rich source for pharmacologically useful antimicrobial peptides (Otero-González et al. 2010).

Ghana possess a coastline of about 550 km with different types of aquatic habitat ranging from deep sea hydrothermal vent to intertidal regions. In addition, a number of freshwater habitats can be found all over the country. These aquatic habitats are home to a wide variety of invertebrates (Ministry of Environment and Science 2002). Research geared towards the isolation of bioactive compounds from organisms thriving in these areas remain largely unexplored. Rather, a lot of attention has being focused on the terrestrial environment with most works dedicated to plant secondary metabolites (Adotey et al. 2012; Asomaning et al. 1999).

This paper reports on the antimicrobial and antioxidant activities of the crude peptide extracts of two molluscs, *Galatea paradoxa* (*G. paradoxa*) and *Patella rustica* (*P. rustica*) obtained from the sea and a freshwater in Ghana respectively. *G. paradoxa* is a bivalve mollusc which belongs to the family Donacidae and is normally constrained to some few rivers in West Africa such as the River Volta in Ghana (Adjei-Boateng et al. 2012; Obirikorang et al. 2013). *P. rustica* is a gastropod belonging to the family Patellidae and can be found on rocky sea shorelines. Peptides from these two molluscs were extracted and tested against nine pathogenic microbes. The antioxidant activities of these extracts were also investigated.

## Results and discussion

### Antimicrobial activity

In this work, the crude peptides from *G. paradoxa* and *P. rustica* were extracted via cold acetone precipitation. Following lyophilization, the crude peptide extracts were reconstituted in 25 % ACN in 0.1 % TFA and used for the antimicrobial assay. Results are summarized in Table 1. Crude peptide extracts from *G. paradoxa* was observed to possess a high bactericidal activity with the highest zone of inhibition of 19.5 mm recorded against *Escherichia coli, Staphylococcus aureus, Bacillus subtilis, Streptococcus pneumoniae* and *Pseudomonas aeruginosa. E. feacalis* exhibited an inhibition zone of 16 mm while *Salmonella typhi* and *Klebseilla pneumoniae* recorded 15 mm. Extracts from *G. paradoxa* however failed to inhibit the growth of the fungus *Candida albicans*. *P. rustica* crude peptide extracts were very effective in inhibiting the growth of the fungus, *C. albicans,* with an inhibition zone of 37 mm. *K. pneumoniae* and *E. feacalis* recorded zones of inhibition of 16.5 mm and 15 mm respectively. The *P. rustica* extracts, however, was ineffective in inhibiting the growth of all other test microorganisms.

**Table 1:** Zone of Inhibition (mm) of extracts of *Galatea paradoxa* and *Patella rustica* against test microorganisms

	*Galatea paradoxa*	*Patella rustica*	Positive control (ciprofloxacin)	Negative control (25 % ACN/0.1 % TFA)
*E. coli*	19.7 ± 0.6	0.0 ± 0.0	36.3 ± 1.2	–
*S. aureus*	20.0 ± 0.0	3.0 ± 0.0	14.7 ± 0.6	–
*B. subtilis*	20.3 ± 0.6	2.0 ± 0.6	19.0 ± 1.0	–
*S. typhi*	15.3 ± 0.6	2.0 ± 1.0	35.3 ± 0.6	–
*E. feacalis*	16.0 ± 1.0	15.0 ± 1.0	34.7 ± 0.6	–
*C. albicans*	0.0 ± 0.0	37.0 ± 1.0	41.0 ± 2.0	–
*K. pneumoniae*	14.7 ± 0.6	16.7 ± 1.15	35.0 ± 0.0	–
*S. pneumoniae*	20.3 ± 0.6	0.0 ± 0.00	15.0 ± 1.7	–
*P. aeruginosa*	20.3 ± 1.5	2.3 ± 1.15	35.3 ± 1.2	–

Values reported as mean ± standard deviation. Mean of three experiments

Zone in mm indicates the distance from the border of the disc to the edge of the clear zone

*ACN* acetonitrile,* TFA* trifluoroacetic acid

The results of the broth dilution test of the various extracts are presented in Table 2. The MICs of *G. paradox* and *P. rustica* towards the various test microorganisms ranged from 20 to 13 mg/mL. *G. paradoxa* extracts recorded the highest MIC of 20 mg/mL towards *C. albicans*, with all other microorganisms giving an MIC of 17 mg/mL. For *P. rustica*, an MIC of 13 mg/mL was recorded for *C. albicans* those of *E. feacalis* and *K. pneumoniae* were both 17 mg/mL. All other microorganisms had an MIC of 20 mg/mL.

**Table 2:** Minimum inhibitory concentrations of extracts of *Galatea paradoxa* and *Patella rustica* against test microorganisms

	Concentrations (mg/mL)													
	*Galatea paradoxa extracts*							*Patella rustica extracts*						
	23	20	17	13	10	7	3	23	20	17	13	10	7	3
*E. coli*	–	–	**	+	+	+	+	–	**	–	–	+	+	+
*S. aureus*	–	–	**	+	+	+	+	–	**	–	–	+	+	+
*B. subtilis*	–	–	**	+	+	+	+	–	**	–	–	+	+	+
*S. typhi*	–	–	**	+	+	+	+	–	**	–	–	+	+	+
*E. feacalis*	–	–	**	+	+	+	+	–	–	**	–	+	+	+
*C. albicans*	–	**	+	+	+	+	+	–	–	–	**	+	+	+
*K. pneumoniae*	–	–	**	+	+	+	+	–	–	**	–	+	+	+
*S. pneumoniae*	–	–	**	+	+	+	+	–	**	–	–	+	+	+
*P. aeruginosa*	–	–	**	+	+	+	+	–	**	–	–	+	+	+

(+), indicates microbial growth; (-), indicates inhibition of microbial growth; (**), indicates MIC

**Fig. 1:**
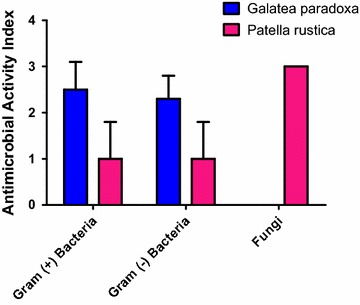
Antimicrobial activity indices of *Galatea paradoxa* and *Patella rustica* crude peptide extracts. AI for each extract was obtained by dividing the sum total of weightages by the total number of test microorganisms. Microorganisms were grouped into Gram positive bacteria, Gram negative bacteria and fungi. Data shown as mean ± SD

In general, the crude peptide extracts of *G. paradoxa* was found to be the most active as evidenced by a higher AI of 2.1. *P. rustica* gave a modest AI of 1.2 indicating weak activity against tested microorganisms (Table 3). *G. paradoxa* extracts demonstrated a higher activity towards both Gram-positive and Gram-negative bacteria with AI of 2.5 and 2.3 respectively. *P. rustica* was much less active, giving an AI of 1 for both Gram-positive and Gram-negative bacteria. However, *P. rustica* extracts proved to be the most active antifungal agent, exhibiting an AI of 3 against fungi compared to *G. paradoxa* which had no activity (AI of 0). Comparison of the activity index of extracts against Gram-positive bacteria, Gram-negative bacteria and fungi is given in Fig. 1.

**Table 3:** Antimicrobial index of crude peptide extracts of *Galatea paradoxa* and *Patella rustica*

	Antimicrobial index
*Galatea paradoxa*	2.1
*Patella rustica*	1.2

### Antioxidant assay

**Fig. 2:**
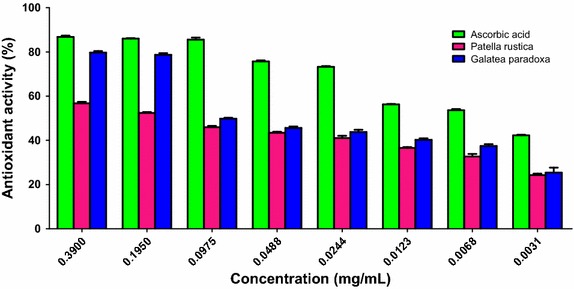
Antioxidant activity of ascorbic acid and extracts of *Galatea paradoxa* and *Patella rustica*. The percent antioxidant activity was obtained at different concentrations of standard and test extracts. The percentage of DPPH scavenging was calculated as follows: DPPH radical scavenging activity (%) = [(Absorbance of control − Absorbance of sample)/Absorbance of control] × 100. Data shown as mean ± SD of three separate experiments

As depicted in Fig. 2, the maximum scavenging ability of *G. paradoxa* was 56.77 % at 0.39 mg/mL and that of *P. rustica* was 79.77 % at the same concentration. Minima of 24.27 and 21.40 % were recorded for *G. paradoxa* and *P. rustica* respectively at 0.0031 mg/mL. Those for the standard ascorbic acid drug were 86.77 and 42.27 % at 0.39 and 0.0031 mg/mL respectively.

## Discussion

In this study, our goal was to identify potential sources of bioactive antimicrobial peptides for further purification. To do this, we examined the antimicrobial activity and antioxidant potential of the crude peptide extracts of two aquatic invertebrates, *G. paradoxa* and *P. rustica*. The results obtained thus far indicates that both molluscs are potentially good sources of antimicrobial peptides. Whereas extracts from *G. paradoxa* displayed impressive activity against both Gram-positive and Gram-negative bacteria, *P. rustica* extracts were much more ruthless towards fungi.

The antimicrobial tests were carried out against 4 Gram-positive bacteria (*S. aureus, B. subtilis, E. feacalis, S. pneumonia*e), 4 Gram-negative bacteria (*E. coli, S. typhi, K. pneumoniae, P. aeruginosa*) and one fungus (*C. albicans*). Extracts of *G. paradoxa* showed markedly high antibacterial inhibition towards *E. coli, S. aureus, B. subtilis, S. pneumonia and P. aeruginosa*. However, no activity was recorded against *C. albicans*. *P. rustica* extracts inhibited the growth of *C. albicans* at concentrations comparable to the positive control (37 mm for *P. rustica* against 40 for the standard drug ciprofloxacin). The extracts from *P. rustica* were however, ineffective towards most of the pathogenic microorganisms used in this study. Based on the zones of inhibition, the AI computed suggested that *G. paradoxa* was a more promising source of antimicrobial peptides than *P. rustica*. This was evidenced by the fact that the AI of *P. rustica* extracts was 1.2 while that of *G. paradoxa* was 2.1. Since AMPs utilize different modes to inhibit Gram-positive bacteria, Gram-negative bacteria and fungi (Sathyan et al. 2014), it was important to compute AI of the various extracts against these classes of microorganisms. It was observed that *G. paradoxa* extracts were over 50 % more active towards both Gram-positive and Gram-negative bacteria. *P. rustica* extracts, though confirmed their high antifungal activity with an AI of 3 towards fungi.

The activities of AMPs from some marine invertebrates have been previously documented. Work carried out on the molluscs, *Mytilis edulis* and *Mytilis galloprovincialis* resulted in the isolation of a number of AMPs (defensins, mytilin, mytimicin and myticin) (Chisholm et al. 2009; Mitta et al. 1999a, b; Miyata et al. 1989). In addition, the antimicrobial activities of the crude peptide extracts of 24 different molluscs have been reported by Sathyan et al. (2014). The antimicrobial activities of crude peptide extracts can be attributed to the presence of a number of different AMPs in the same sample. These AMPS could act in synergy or antagonistically. Isolation and characterization of these AMPs will go a long way in shedding light on this phenomenon.

The goal of this study was to characterize the antimicrobial and antioxidant properties of the crude peptide extracts of two Ghanaian molluscs. Review of literature has not shown any antimicrobial activity of the crude peptide extracts of *P. rustica* and *G. paradoxa*. Interestingly, crude peptide extracts that exhibit good antifungal activities are usually ineffective against any form of bacteria (Sathyan et al. 2014). The *P. rustica* extracts in this work followed such trend, displaying poor antibacterial activity but potent antifungal properties. This could be indicative of a different mode of action of AMPs towards different microorganisms (Sathyan et al. 2014; der Van Weerden et al. 2013).

While peptides have been documented to possess significant antioxidant activities (Suetsuna 2000; Mendis et al. 2005; Jung et al. 2004), most work on AMPs from marine molluscs do not consider this. In light of this, we investigated the antioxidant capacity of the crude peptide extracts from both *G. paradoxa* and *P. rustica*. When compared to the standard ascorbic acid, the percent antioxidant activity of the crude peptide extracts were lower. Both *G. paradoxa* and *P. rustica* extracts exhibited comparable activity at low concentrations. However, at high concentrations, the *P. rustica* extracts did a better job at scavenging the DPPH radicals. The lower percent antioxidant activities recorded might be due to the fact that the extracts used were in their crude, unpurified state. It is however difficult to draw any correlation between antioxidant capacity and antimicrobial activity based on these results.

## Conclusions

In the present study, crude peptides extracted from two Ghanaian mollusks exhibited impressive antimicrobial activity against all nine pathogenic microorganisms tested. *G. paradoxa* extracts were found to be promising source of highly potent antibacterial agents whereas the *P. rustica* extracts were better antifungal agents. Both extracts demonstrated appreciable antioxidant activity that could be harnessed. The study confirms molluscs as potentially rich sources of antimicrobial and antioxidant peptides. Future work could focus on the isolation, purification and characterization of the bioactive components.

## Methods

### Sample collection

*Galatea paradoxa* and *P. rustica* samples used in this study were handpicked from their various habitats in February 2015. *G. paradoxa* was obtained from River Volta in the Volta Region of Ghana. *P. rustica* was collected from rocks in the sea at the Labadi Beach in the Greater Accra Region of Ghana. Samples were kept on ice and transported to the laboratory for analysis. Authentication of *G. paradoxa* and *P. rustica* samples used in the study were done at the Department of Fisheries and Marine Sciences, Kwame Nkrumah University of Science and Technology (KNUST), Kumasi—Ghana.

### Extraction

The shells of the two species were removed and the tissue chopped into smaller pieces. The body tissue was then homogenized with five folds (60 mL) w/v 10 % acetic acid and kept for 12 h at 4 °C. The extract obtained was centrifuged for 15 min at 4 °C and 3000 rpm (TD3 Tabletop centrifuge, China) and the supernatant collected. Ice cold acetone (25 mL) was then added and the entire mixture kept at 4 °C for 12 h. The precipitate formed was collected and freeze dried (Labconco, Kansas City) for 1 h. It was then reconstituted in 25 % acetonitrile(ACN) prepared in 0.1 % trifluoroacetic acid (TFA) (Sathyan et al. 2014).

### Antimicrobial assay

#### Microbial cultures

Eight strains of bacteria and one fungal strain were used to assess the antimicrobial properties of the crude extracts. The Gram negative bacteria *Escherichia coli* (*E. coli*), *Salmonella typhi* (*S. typhi*)*, Klebseilla pneumoniae* (*K. pneumoniae*) *and Pseudomonas aeruginosa* (*P. aureginosa*) and Gram positive bacteria *Staphylococcus aureus* (*S. aureus*), *Bacillus subtilis* (*B. subtilis*), *Enterococcus feacalis* (*E. feacalis*) *and Streptococcus pneumoniae* (*S. pneumoniae*) were used as test microorganisms. The fungal strain used for the antimicrobial assay was *C. albicans* (*C. Albicans*). All the microbial strains were obtained from the Department of Pharmaceutical Chemistry, College of Health Science, KNUST, Kumasi, Ghana.

#### Inoculum preparation

Sabouraud growth media was prepared and sterilized in an autoclave at 121 °C for 15 min. All 9 microbes were individually incubated at 37 °C for 18 h and 72 h in case of the fungus.

#### Diffusion well assay

The antimicrobial activities of the crude extracts were evaluated by diffusion well assay. The nine microorganisms were cultured on nine different agar plates with three wells in each plate. 20 μL portions of each extract was pipetted into each well. Ciprofloxacin was used as the standard antibiotic (positive control) while 0.1 % TFA in 25 % ACN was used as negative control. The plates were incubated at 30 °C for 24 h. The zone of inhibition was measured in millimeters (mm) as the distance from the border of the disc to the edge of the clear zone. The assay was repeated three times and the averages of the three experiments taken.

#### Minimum inhibitory concentration

The minimum inhibitory concentration (MIC) was determined by broth micro dilution assay. Nutrient broth, microorganisms and the extract was mixed up to a final volume of 200 μL using a 96 well microtiter plate. Serial dilutions of the extracts were poured into subsequent wells. Across the rows for each microtiter plate, 10 μL of the nine different test organisms were added. This was followed by addition of 100 μL of nutrient broth to each well. Finally, sterile water was added to top up to the 200 μL mark. The plates were then covered and incubated at 30 °C for 24 h. After 24 h the plates were removed and a solution of 3-(4,5-dimethylthiazol-2-yl)-2,5-diphenyltetrazolium bromide (MTT) was added to each well. The wells that changed colour to violet are indicative of the growth of microorganisms, whiles those that remains unchanged indicated inhibition of microbial growth by the crude extracts.

#### Antimicrobial activity index

For each crude peptide extract, the antimicrobial index (AI) were calculated as the average of the antimicrobial activity obtained against all test microorganisms. Weightages were assigned to the activity of extracts against each test organism as follows; a weightage of one (1) for zone of inhibition up to 10 mm, two (2) for zone of inhibition ranging from 11 to 20 mm, three (3) if the zone of inhibition was greater than 20 mm and zero (0) for no antimicrobial activity. The AI was obtained by dividing the sum total of weightages obtained by each crude extract by the total number of test microorganisms. Separate AI was calculated for Gram positive bacteria, Gram negative bacteria and fungi to compare the activity of the two extracts (Ghosh et al. 2008; Sathyan et al. 2014).

#### Antioxidant activity assay

The free radical scavenging capacity of the extracts was determined using the 2, 2-diphenyl-1-picrylhydrazyl (DPPH) assay. Serial dilutions of extracts were prepared in different test tubes and freshly prepared DPPH solution added to a final volume of 3 mL. After 30 min, the absorbance was read at 517 nm using a UV–Visible spectrophotometer (Perkin Elmer Lambda 35). Ascorbic acid was used as reference standard. Control sample was prepared containing the same volume without any extracts. Absorbance of freshly prepared DPPH was also read. Methanol was used as the blank.

## Abbreviations

ACN: acetonitrile
AI: antimicrobial index
AMP: antimicrobial peptide
DPPH: 2,2-diphenyl-1-picrylhydrazyl
EVD: Ebola viral disease
MIC: minimum inhibitory concentration
MTT: 3-(4,5-dimethylthiazol-2-yl)-2,5-diphenyltetrazolium bromide
SD: standard deviation
TFA: trifluoroacetic acid
